# Hydatid cyst of the heart as a rare cause of arrhythmia: A case report and review of published reports

**DOI:** 10.1016/j.hrcr.2022.04.004

**Published:** 2022-04-09

**Authors:** Zhenyu Dong, Muyassar Yusup, Yanmei Lu, Baopeng Tang

**Affiliations:** Department of Cardiac Pacing and Electrophysiology and Xinjiang Key Laboratory of Cardiac Electrophysiology and Remodeling, The First Affiliated Hospital of Xinjiang Medical University, Urumqi, China

**Keywords:** Cardiac hydatid, Hydatid cyst, Arrhythmia, Case report, Literature review

**Key Teaching Points**

• The most frequently reported arrhythmia related to cardiac hydatid is ventricular tachycardia (VT), followed by high-grade atrioventricular block.
• The most common main complaint of VT is palpitations while that of high-grade atrioventricular block is syncope.
• Surgery is the first choice for the treatment of cardiac hydatid cysts, and the chemotherapy using albendazole and/or mebendazole is used as an adjunct to surgical treatment to prevent the recurrence of cysts.

## Introduction

Hydatid cysts result from infection by either *Echinococcus granulosus* or *Echinococcus multilocularis*. Cystic echinococcosis has traditionally been most common in countries where large numbers of sheep and cattle are raised; however, well-developed transportation has made this condition a serious global health problem. According to the World Health Organization, more than 1 million people are affected by hydatid disease at any time.^1^^,^^2^

The heart is a very rare parasitic location for hydatid. Clinical manifestations of the hydatid cyst of the heart are atypical and of varied presentation, from completely asymptomatic forms to those presenting as severe cardiac failure. Arrhythmia caused by cardiac hydatid cyst has a high fatality rate, but it is extremely rare, so there is a great possibility of missed diagnosis, especially for doctors in hospitals not located in pastoral areas. This existing difficulty in the preoperative recognition of this clinical entity has induced us to report our case, which is described below. We also conducted a literature review of previously reported patients with cardiac hydatid and arrhythmia, in order to facilitate clinical diagnosis and treatment.

## Case report

**Figure 1:**
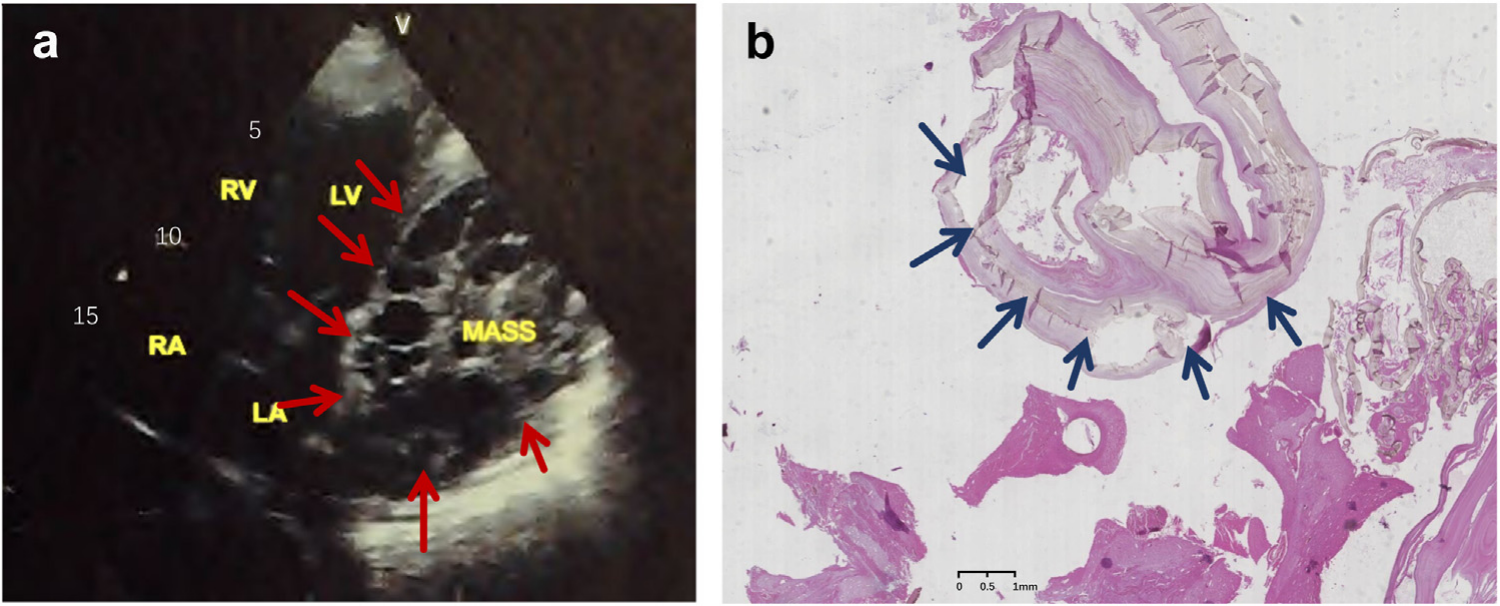
**a:** Preoperative echocardiogram; red arrow points to the hydatid cyst. **b:** Pathologic picture; blue arrow points to the endocyst. LA = left atrium; LV = left ventricle; RA = right atrium; RV = right ventricle.

A man in his 40s was referred to our hospital with intermittent chest pain and palpitations accompanied by 1 episode of syncope, which had been present for 1 day. The past medical history of the patient included hepatic hydatidectomy. The electrocardiogram of the patient in other hospitals showed paroxysmal ventricular tachycardia (VT). The echocardiography after admission showed that there was a huge mass of about 11 × 9.8 × 7.5 cm in the myocardium of the basal segment of the posterior left ventricle, and the myocardium was thin (Figure 1a). The envelope of the mass is complete and clear, without breaking through the pericardial visceral layer. Combining the patient’s medical history, symptoms, and examination results, we consider that the patient was diagnosed with cardiac echinococcosis, and VT is caused by this primary disease. The patient was advised to go to the Department of Cardiac Surgery for hydatidectomy. After the patient agreed, he was transferred to the Department of Cardiac Surgery for treatment. The operation was successful. The pathologic image of the excised hydatid tissue is shown in Figure 1b. The patient’s VT did not reappear within 3 months after the operation.

## Literature review

### Search strategy

We carried out a literature review of arrhythmia occurring with cardiac hydatid according to the PRISMA guidelines and recommendations.^3^ On September 20, 2021, we performed a computerized PubMed database search to identify peer-reviewed articles previously published since the database inception. Keywords searched were “Cardiac hydatid,” “Cardiac echinococcosis,” and “arrhythmia.”

### Study selection and data extraction

The titles and abstracts were independently systematically screened by 2 authors (Z.D. and M.Y). If duplicate articles or patients were found, only the most recent reports were included. Full-text articles were then further reviewed. Inclusion criteria were original text discussing the occurrence of arrhythmia in cardiac echinococcosis. We included any case reports, case series, retrospective studies, and prospective reports. Exclusion criteria were hydatid disease that does not occur in the heart, hydatid disease without arrhythmia, and non-English language.

Two authors (Z.D. and M.Y.) independently recorded data, and any discrepancies were reviewed in conference. The variables recorded were sex, age, date published, type of arrhythmia, initial presenting symptoms, location and size of hydatid cyst, treatment, and prognosis. All raw data were recorded, but the data were cleaned as follows for statistical analysis: (1) we considered that cardiac hydatid is a hydatid located in the heart cavity or myocardium or pericardium; (2) because the volume of the hydatid cyst is described differently in different articles, we analyze the data of the longest diameter of the cyst.

### Statistical analysis

Descriptive statistics were reported using the entire sample size. Unless the absence of a particular variable was explicitly stated, we considered it null and excluded it from inferential statistics. The correlation between diameter of hydatid and mortality rate are analyzed by the point-biserial correlation method. Analyses were performed using SPSS (version 25.0; SPSS Institute Inc, Armonk, NY). Mean (range ± standard deviation) values are reported. *P* ≤ .05 was considered statistically significant.

## Results

### Study characteristics

**Figure 2:**
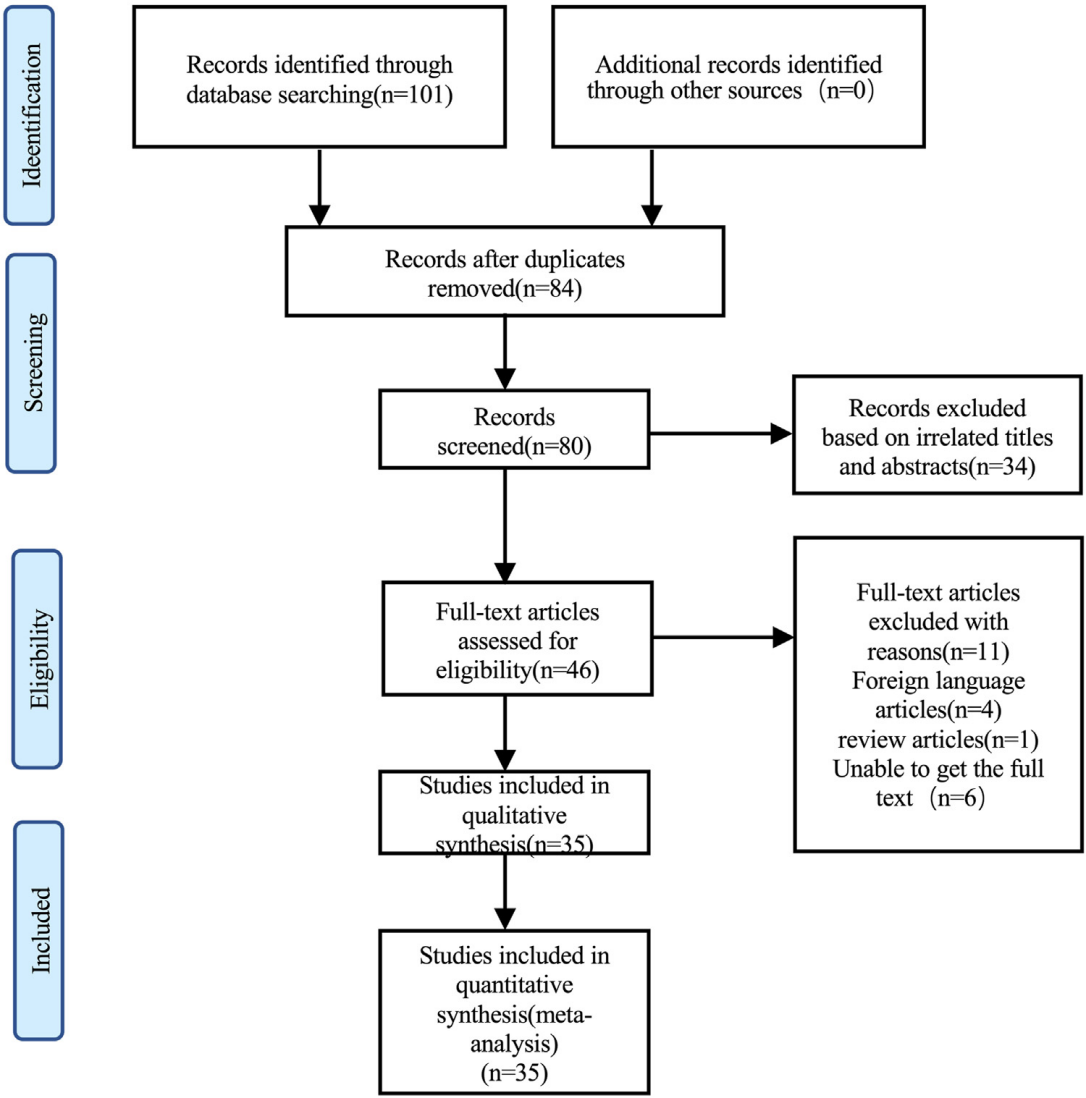
Document screening flow chart.

The initial database search yielded 101 articles. Further screening resulted in 46 articles that were accessed for inclusion and exclusion criteria. Ultimately 35 articles met inclusion criteria, with a resultant 41 cases. These 41 cases were combined with our case, for a total sample size of n = 42 for statistical analysis (Figure 2). Raw data results of the literature review are shown in Supplemental Table 1.

### Risk-of-bias assessment

All articles were susceptible to inherent publication biases that result from preferential publishing of cases with unusual results. Studies were potentially biased in terms of selective outcome and incomplete data reporting, in that all pertinent surgical events or clinical information may not have been described. This led to significant heterogeneity in the type and amount of information that was analyzed in our review. All studies included in our analysis were either level 4 evidence case reports or case series, which limits the strength of our conclusions.

### General condition of the patient

The literature that we can find about cardiac hydatid with arrhythmia was first published in 1951, reported by Ghanem and Darwish.^4^ Among the reported cases, most patients (24/37, 64.9%) were male. The mean age was 35.7 years old (range 1–79, ± 20.1 years). For patients with cardiac arrhythmia caused by echinococcosis, symptom-related data of 28 patients were reported, of which patients with no symptoms accounted for 10.7% (3/28), while the most common initial presenting symptom was palpitations (42.9%, 12/28). According to the frequency of occurrence, the order of other symptoms is dyspnea (28.6%, 8/28), syncope or presyncope symptoms (amaurosis, etc) (21.4%, 6/28), fatigue (17.9%, 5/28), fever (14.3%, 4/28), dizziness (10.7%, 3/28), and digestive system symptoms (10.7%, 3/28). The most common symptom of cardiac hydatid combined with VT is palpitations (54.5%, 6/11), and the most common symptom of cardiac hydatid combined with third-degree atrioventricular block (AVB) is syncope or presyncope symptoms (75%, 3/4).

### Details of heart hydatid

For the reported cardiac hydatid patients with arrhythmia, the hydatid diameter is 5.6 cm (range 1.0–11.0 cm, ± 2.6 cm), which basically conforms to the normal distribution. The diameter of hydatid is not related to the survival rate of the patient (*P* = .806). The most common location of cardiac hydatid is the interventricular septum (IVS) (53.8%, 21/39); other common locations are left atrium (12.8%, 5/39), left ventricle (20.5%, 8/39), and the pericardial sac (5.1%, 2/39). For patients with VT and cardiac hydatid, the most common location of hydatid is in the IVS (50%, 6/12), followed by the ventricle (41.7%, 5/12). For patients with third-degree AVB and cardiac hydatid, the most common location of hydatid is in the IVS (88%, 7/8). The mortality rate of hydatid implanted in the IVS was 22.2% (2/18) and the mortality rate of hydatid implanted in the ventricle was 33.3% (3/9).

### Arrhythmia and treatment

For patients with cardiac hydatid, the most frequently reported arrhythmia is VT. The diameter of the cardiac hydatid that causes VT is 6.3 cm (1–11 cm, ± 2.6 cm), and it is most often located in the IVS. The second most commonly reported arrhythmia is third-degree AVB. The diameter of the heart hydatid causing third-degree AVB is 4.9 cm (1.9–8 cm, ± 2.7 cm), and the most common location is also the IVS.

The initial interventions performed were surgery. A total of 52.6% (18/38) of patients with cardiac hydatid and arrhythmia underwent hydatid removal. Hydatid removal surgery is significantly related to the survival rate of patients (*P* < .05). There are 18 cases reporting on the order of surgery or antiparasitic drugs. Among 3 cases, hydatid removal was performed first, and the patient mortality rate was 100%; in 15 cases the patients were first treated with antiparasitic drugs, and the patient mortality rate was 21.4%.

## Discussion

In this article, we reported a case of cardiac hydatid with VT. The main complaints of the patient were chest pain and palpitations when admitted to the hospital. This case showed a rare cause of arrhythmia, which is easy to be misdiagnosed or missed. We also conducted a literature review of cardiac hydatid combined with arrhythmia. As far as we know, so far no studies have reviewed cardiac arrhythmias caused by echinococcosis, most of which are reported in the form of case reports. According to the results of our analysis, the mortality rate was 32.4%. On the one hand, the high mortality rate reflects the great harm of cardiac hydatid combined with arrhythmia, which requires clinicians to attach great importance to early identification, diagnosis, and treatment. On the other hand, the high mortality rate suggests that the treatment of arrhythmia itself is also very important—for example, implantable cardiac defibrillators (ICD) and pacemakers. The most frequently reported arrhythmia related to cardiac hydatid is VT, followed by high-grade AVB. The most common main complaint of VT is palpitations while that of high-grade AVB is syncope, which is consistent with the symptoms of idiopathic arrhythmia. Other symptoms include dyspnea and fatigue. The most common parasitic location is the IVS, which is the substrate of the heart’s electrical conduction. The size of cardiac hydatid varies, and the size of the cyst does not seem to be related to the mortality rate.

Previous research suggests that hydatid cysts were found in 4 different sites of the heart: 47% in the left ventricle, 26% in the pericardium, 16% in the right ventricle, and 11% in the IVS.^5^ Another study suggests that hydatid cysts are most commonly located in the free wall of the left ventricle. IVS involvement is observed in 4% of cases.^6^ However, our results are inconsistent with previous studies on cardiac hydatid, which may be owing to the particularity of cardiac hydatid that causes arrhythmia. This proves the causation between arrhythmia and the cysts from another aspect. A study by Yasim and colleagues^7^ suggests that most patients are asymptomatic, but in our study the most common symptom is palpitations, which may be related to the arrhythmia of the population we included.

Up to 80% of patients have involvement of a single organ, and isolated cysts are predominantly localized to the liver or lungs. Cardiac infections occur in 0.5%–2% of all human echinococcosis cases.^8^ Cardiac arrhythmias occur when the electrical bundles of the heart are mechanically affected by the cysts; in our study, it is mostly in the IVS. We believe that it is because hydatid affects the conduction system, which leads to the occurrence of VT and third-degree AVB. In fact, after hydatid removal, the arrhythmia disappeared in most cases.

### Diagnosis

Cardiac hydatid is relatively rare and cardiac hydatid cysts grow slowly, have a long incubation period, and have atypical clinical symptoms, which all make timely diagnosis difficult, and the condition is easy to miss and misdiagnose. Research indicates that about 10% of the annual cases are not officially diagnosed.^9^ Cardiac tumors and thrombosis (if the patient has a history of valve disease) should be differentially diagnosed, especially if the patient comes from an endemic area of hydatid disease and has a history of close contact with dogs, sheep, and other animals. Serum immunological examination has high diagnostic value and helps prevent misdiagnosis. However, laboratory tests are usually ambiguous and the sensitivity and specificity of various immunological diagnostic methods are different, and the simultaneous application can increase the sensitivity and specificity. At the same time, the location and structure of the cardiac hydatid cyst will also affect the results of serological examinations. Serum immunological examinations should be performed multiple times at different stages of the pericardial disease course to prevent misdiagnosis and missed diagnosis. Echocardiography, computed tomography, and magnetic resonance imaging are useful in the diagnosis and location of cardiac echinococcosis. In addition, when electrocardiogram abnormalities are suggestive of myocardial ischemia the suspicion should be very strong indeed.

### Treatment

Surgery is the first choice for the treatment of cardiac hydatid cysts, and chemotherapy using albendazole and/or mebendazole is used as an adjunct to surgical treatment to prevent the recurrence of cysts. Technical challenges from a surgical perspective included total removal of the cyst without disseminating its contents, as well as a potentially complex reconstruction of the IVS. Some researchers think presurgical medical management is essential to reduce the activity of the target pathology and to reduce the likelihood of echinococcus dissemination.^10^ However, early application of the chemotherapy may cause the cyst to rupture. We believe that unless the patient’s condition is too bad, the cyst is too large for surgery, or the operation has too much damage to the IVS, it is recommended to perform hydatidectomy as soon as possible. It is worth pointing out that radiofrequency ablation, pacemaker implantation, and ICD are also very important.

### Study limitations

Because there is a very significant publication bias, it cannot be considered that cardiac hydatid is prone to VT, AVB, etc. Most patients in whom VT and AVB is encountered in clinical practice should not be considered as having cardiac hydatid. Like the proverb that many clinicians are familiar with, when you hear the sound of horseshoes, you should not think of zebras. But for those who come from the epidemic areas or have previously been infected with hydatid (liver hydatid, etc) or those in special occupations such as chefs, cardiac hydatid should be considered as a possible cause.

## Conclusion

In conclusion, we present a case illustrative of arrhythmia related to cardiac hydatid cyst. Results from our literature review are limited by heterogeneity but showed this disease is much more common in males and typically occurs in young adults. The pathophysiology behind this phenomenon is not fully understood but probably exists on the blocked electrical conduction system. Distinguishing cardiac hydatid cyst from idiopathic arrhythmia is challenging. We are reporting this review to underline that cardiac hydatid should be considered as a differential diagnosis in patients who live in endemic regions presenting with unexplained cardiac symptoms. First-line treatment includes surgery and antiparasitic drugs, but ICD and pacemakers are also critical to prognosis. Further research is needed to help clinicians avoid this uncommon but serious complication.

## References

[1] Tufekcioglu O., Birincioglu C.L., Arda K., Fansa I., Saritas A., Karahan M. (2007). Echocardiography findings in 16 cases of cardiac echinococcosis: proposal for a new classification system. J Am Soc Echocardiogr.

[2] Lu Y.M., Zhang L., Xing Q. (2019). Ventricular tachycardia as the initial symptom of cardiac hydatidosis. Chin Med J (Engl).

[3] Liberati A., Altman D.G., Tetzlaff J. (2009). The PRISMA statement for reporting systematic reviews and meta-analyses of studies that evaluate healthcare interventions: explanation and elaboration. BMJ.

[4] Ghanem M.H., Darwish A.E. (1951). Hydatid heart disease with paroxysmal tachycardia. Br Heart J.

[5] Noaman H., Rawaf S., Majeed A., Salmasi A.M. (2017). Hydatid cyst of the heart. Angiology.

[6] Carità P., Verdecchia M., Ferro G. (2016). Multimodality imaging in cardiac echinococcosis for diagnosis and follow-up of an untreatable cyst. Int J Cardiol.

[7] Yasim A., Ustunsoy H., Gokaslan G., Hafız E., Arslanoglu Y. (2017). Cardiac echinococcosis: a single-centre study with 25 patients. Heart Lung Circ.

[8] Kammerer W.S., Schantz P.M. (1993). Echinococcal disease. Infect Dis Clin North Am.

[9] Wen H., Vuitton L., Tuxun T. (2019). Echinococcosis: advances in the 21st century. Clin Microbiol Rev.

[10] Separovic Hanzevacki J., Gasparovic H., Reskovic Luksic V., Ostojic Z., Biocina B. (2018). Staged management of a giant cardiac hydatid cyst: a case report. BMC Infect Dis.

