# Supplementary material for: Hydatid cyst of the heart as a rare cause of arrhythmia: A case report and review of published reports
Source: HeartRhythm Case Rep. 2022 Apr 9;8(6):458–62. doi: 10.1016/j.hrcr.2022.04.004 (PMC9237349; doi:10.1016/j.hrcr.2022.04.004)
Supplement: Supplemental Table 1 [file mmc1.pdf]

Number	Die or Live	Surgery	Which first(surgery or Antiparasitic drugs)	Titile	Year	Complaint	Gender	Age	Location	diameter	Arrhythmia
1	live	yes	none	Hydatid Cysts of the Ventricular Septum of the Heart A Study Based on Two Personal Cases and Forty-One Observations in the Literature*	1964	none	male	26	middle part interventricu lar [illegible]	6.0	right bundle branch block.
2	live	yes	none	Ventricular tachycardia as the initial symptom of cardiac hydatidosis	2019	repeated episodes of having palpitations, accompanied with cold sweats, amaurosis, vomiting, and limb weakness in the previous 2 months	female	44	the posterior apex of the [illegible]	6.2	ventricular tachycardia. T segment depression and T wave inversion
3	live	none	drug	Hydatid disease and the heart	2021	history of on and off low grade fever for 8days followed by progressive shortness of breath associated with orthopnea for 5 days.	male	28	the basal interventricu lar septum, [illegible]	3.3	sinus tachycardia.
4	live	yes	none	Hydatid disease of the heart a report on 2 cases treated surgically	1962	frequent palpitation, precardial distress, and sometimes striking pains, which spread to the ncck and the back, of a total duration of 3 months.	female	31	inside the pericardial sac anterior [illegible]	10.0	sinus rhythm with occasional atrial extra-systoles, and T-waves were depressed in all the three leads
5	die	no	drug	An Incidental Finding of Heart Echinococcosis in a Patient with Infective Endocarditis: a Case Report	2017	10-day history of persistent fever as high as 40°C, accompanied by fatigue and loss of appetite	none	77	at the upper part of the left atrium	3.5	permanent atrial fibrillation
6	die	no	none	Hydatid heart disease with paroxysmal tachycardia.	1951	palpitation,cough,and dyspncea of three days'duration.	male	22	at the base of the left ventricle	5.0	paroxysmal supraventicular tachycardia
7	die	no	none	Cardiovascular echinococcosis	1969	increasingly severe nausea, vomiting, and anorexia	male	35	wall of the left pulmonary [illegible]	4.0	incomplete right bundle branch block,
8	live	yes	none	Restoration of atrioventricular conduction after surgical removal of a hydatid cyst of the interventricular septum	1987	dizziness of recent onset	male	31	located in the infundibular [illegible]	8.0	third-degree AVblock with a ventricular rate of 50 beats/min.
9	live	no	drug	Left atrial mobile hydatid cyst mimicking left atrial myxoma and mitral stenosis and causing heart failure and arrhythmia	2007	dyspnea, fatigue, intermittent palpitations lasting for a long time	female	78	left atrial	none	paroxysmal atrial fibrillation
10	none	none	none	Interventricular hydatid cyst with atrioventricular block: A case report	2003	none	female	21	interventricu lar septum	2.5	III AVB
11	die	no	none	A giant cardiac hydatid cyst presenting with chest pain and ventricular tachycardia in a pregnant woman undergoing cesarean section	2016	asymptomatic	female	33	in the interventricu lar septum [illegible]	3.6	sustained ventricular tachycardia
12	live	yes	none	Cardiac hydatid cyst revealed by complete heart block	2000	syncope	female	28	in the wall of the interventricu [illegible]	2.7	III AVB
13	live	yes	none	Echocardiographic differentiation of a cystic and a solid tumor of the heart	1977	none	male	8	ventricular septum	none	right axis deviation and a pattern of right bundle branch block
14	die	yes	none	above	1977	none	none	4-day	arise from the free left ventricular [illegible]	none	right ventricular conduction delay
15	live	no	drug	Right ventricular hydatid cyst presented as tachyarrhythmia	2019	syncope	male	57	in the right ventricular apical [illegible]	1.0	ventricular tachycardia
16	live	yes	none	Paroxysmal ventricular tachycardia due to interventricular hydatid cyst	2002	complaining of short-lasting attacks of palpitations and lightheadedness that started 1 week	male	35	the left ventricular cavity in the [illegible]	6.0	short-lasting ventricular tachycardia
17	live	yes	drug	Interventricular septal hydatid cyst presenting as complete heart block	1996	Stokes-Adams attacks, which he had had for 18 months.	male	25	middle third of the interventricu [illegible]	8.0	complete heart block.
18	none	none	none	Hydatid Cyst of the Heart	2017	none	none	none	the interventricu lar septum	none	fast atrial fibrillation
19	none	none	none	above		none	none	none	the interventricu lar septum	none	multifocal ventricular ectopic beats
20	live	yes	none	Hydatid cyst of the cardiac interventricular septum with complete atrioventricular block: A case report from Pakistan	2012	recurrent syncopal	male	30	none	none	III avb
21	live	yes	surgery	Ventricular tachycardia: A complication of an intramyocardial echinococcal cyst	2000	palpitations, dyspnea, and near-syncope 1day	male	23	on the dorsal aspect of [illegible]	8.0	ventricular tachycardia
22	live	yes	none	Hydatid cyst of interventricular septum causing left anterior hemiblock.	1971	asymptomatic	male	11	the interventricu lar septum.	10.0	left anterior hemiblock with right bundle-branch block
23	live	yes	surgery	Cardiac echinococcus complicated by ventricular tachycardia	2001	sudden onset of chest discomfort followed by presyncope	male	32	the apex of the left ventricle	4.0	sustained ventricular tachy- cardia
24	live	yes	none	Ventricular tachycardia revealing a hydatid cyst	1987	none	male	71	the LV apex, separated [illegible]	6.0	Ventricular tachycardia/right bundle branch block
25	live	yes	drug	Surgical treatment of an Echinococcus cyst of the interventricular septum complicated by septic endocarditis, complete atrioventricular block, and rupture into the valsalva sinus	2010	weakness and a high temperature (40°C) for the last 2 weeks	male	40	the interventricu lar septum	2.9	IIIAVB
26	live	yes	drug	Staged management of a giant cardiac hydatid cyst: A case report	2018	fatigue and palpitations	male	21	the basal and mid part of the [illegible]	8.2	non-sustained ventricular tachycar- dia
27	live	no	drug	Intracardiac masses in young Africans: Case reports and a brief review of the literature	2012	recent onset of palpitations, chest pain, fever, congestive heart failure but no neurological signs.	female	20	the interventricu lar septum	none	First-degree atrioventricular block alternating with periods of type II second- degree block was present on the electrocardiogram.
28	live	no	drug	above	2012	conscious, with flaccid left hemiplegia and mild cyanosis	female	14	left atrial	none	sinus tachycardia
29	live	yes	surgery	Sustained Ventricular Tachycardia as the First Manifestation of Hydatid Cyst Located in the Interventricular Septum	2015	palpitation and dizziness of 20-minute duration	male	17	the basal and middle portion of [illegible]	6.6	ventricular tachycardia
30	die	no	drug	Complete atrio-ventricular block due to cardiac echinococcosis	1979	none	male	33	the septum	8.0	a third degree A-V block, with QRS complexes showing left bundle branch block pattern (QRS duration: 0.13 sec)
31	live	yes	drug	Hydatid cyst of the interventricular septum presenting as supraventricular tachycardia	2012	palpitation and dyspnea	male	55	in the left ventricular side of [illegible]	2.0	supraventricular tachycardia
32	live	no	drug	Complete heart block caused by cardiac echinococcosis and successfully treated with albendazole	1997	none	male	40	on the membranou sportion	1.9	III AVB(35-40)
33	none	no	drug	Pan Cardiac Hydatid Cyst	2013	progressive dyspnea, atypical chest pain and fatigue for two weeks	male	46	two cyst in the left and right atrium [illegible]	none	sinüs tachycardia
34	live	yes	none	Tachycardie ventriculaire recidivante par kyste hydatique du coeur. guerison apres resection chirurgicale. rapport d'un cas	1975	asymptomatic	female	66	none	none	ventricular tachycardia
35	die	no	drug	Ventricular Tachycardia and the Cystic Heart: A Case Report	2020	3-day history of worsening cough, shortness of breath, and dyspnea	female	79	interventricu lar septum and [illegible]	8.2	intermittent monomorphic ventricular tachycardia
36	live	pacemaker but no operation	drug	Extremely rare cardiac involvement: Recurrent pericardial hydatid cyst	2012	exertional dyspnea, palpitation and presyncope attacks lasting for a period of 6 months	male	72	in the pericardium of the left [illegible]	3.7	atrial fibrillation
37	die	no	none	Case Report: Sudden death related to unrecognized cardiac hydatid cyst [version 2; peer review: 2 approved]	2020	none	male	26	occupying half the volume of [illegible]	5.0	scd
38	die	no	none	Sudden Death due to an Unrecognized Cardiac Hydatid Cyst	2011	none	male	28	left ventricle	8.0	scd
39	die	no	none	Sudden death due to an unrecognized cardiac hydatid cyst:: Three Medicolegal Autopsy Cases	2006	none	female	16	none	4.5	scd
40	die	no	none	above	2006	none	female	39	the atria and ventricles	7.0	scd
41	die	no	none	above	2006	none	female	28	in the interventricu lar septum	4.0	scd
42	live	yes	none	myself	2021	palpitations、chest pains、syncope	male	52	left venricular	11.0	vt
